# Supplementary material for: Moving through Motherhood: Involving the Public in Research to Inform Physical Activity Promotion throughout Pregnancy and Beyond
Source: Int J Environ Res Public Health. 2021 Apr 23;18(9):4482. doi: 10.3390/ijerph18094482 (PMC8122923; doi:10.3390/ijerph18094482)
Supplement: Supplementary file 1 [file ijerph-18-04482-s001.zip › Supplementary file S1 MTM Survey Final Version.pdf]

Moving Through Motherhood

A survey about physical activity during pregnancy

We would like to invite you to take part in a survey regarding your views and/or experiences of physical activity during pregnancy, and the information that you may or may not have received around physical activity during pregnancy.

You have been invited to participate as you are at least 18 years of age, and you may have experience of being pregnant and/or being a mother. We would still like to hear from you even if you feel you are/were not physically active during your pregnancy.

Before you take part it is important that you understand what is involved.

- The survey will take **10-15 minutes** to complete.
- There are **no right or wrong answers** to any of the questions, we are interested in your personal views and experiences.
- The survey is **entirely anonymous**, we will not ask for or collect ANY personal information, or ANY information that could be used to identify you.

- Taking part is **entirely voluntary**, if you choose to stop the survey at any point while you are completing it, your data will be removed.

The survey has been developed as part of a collaboration between the Universities of Exeter, Bath and Leicester and has been reviewed and approved by the University of Exeter Sport and Health Sciences Research Ethics committee (Reference 171206-A-03).

Thank you for taking the time to read this information. If you have any questions or concerns regarding any aspect of this survey please feel free to contact Dr Victoria Salmon (V.Salmon@exeter.ac.uk) or Dr Richard Pulsford (R.Pulsford@exeter.ac.uk).

If you are happy to take part please select '**Start Survey'** below. By selecting 'Start Survey' you are confirming that you are at least 18 years of age, have read and understood the survey information described above, and agree to participate in this survey. ✱ *Required*

○ Start Survey

About you

Age (years)

- 18 - 24
- 25 - 34
- 35 - 44
- 45 - 54
- 55 - 64
- 65+

Where do you live? ✱ *Required*

- Scotland
- Wales
- Northen Ireland
- England
- Other

If you selected Other, please specify:

If you live in England, in what region do you live?

Which of these best describes the highest level of education you have completed?

- Secondary education
- College/further education
- Trade/technical/vocational training
- Undergraduate degree
- Post graduate degree

What is your ethnic group?

- White
- Mixed / multiple ethnic groups
- Asian / Asian British
- Black / African / Caribbean / Black British
- Prefer not to say
- Other ethnic group

If you selected Other, please specify:

Are you currently:

- Pregnant
- A Mother
- Both
- Neither
- Prefer not to say

How many children do you have?

What age is your youngest child (years)?

- 0 - 2
- 3 - 5
- 6- 10
- 11 - 15
- 16+
- Not applicable

Have you experienced any of the following during pregnancy? Please select all that apply.

- Back pain
- Pelvic pain
- Leaking (urine/faeces)
- Tiredness
- Varicose veins
- Tummy muscles separation
- Feeling sick/nausea
- Vomiting
- Other, please specifiy
- No symptoms experienced

If you selected Other, please specify:

Information about physical activity

Physical activity includes all forms of activity, such as everyday walking or cycling to get from A to B, active play, work-related activity, active recreation (such as working out in a gym), dancing, gardening or playing active games, as well as organised and competitive sport.

Have you ever received or read information about being physically active during pregnancy?

- Yes
- No

Information you received

Where did you receive this information? Tick as many as you like.

- ☐ Online Forum
- ☐ Website
- ☐ Poster
- ☐ Diagram/Infographic
- ☐ Fitness Professional
- ☐ Family member
- ☐ Friends
- ☐ Leisure centre
- ☐ Magazine
- ☐ Leaflet
- ☐ Midwife & Health Professional
- ☐ Antenatal / Parentcraft classes
- ☐ GP Surgery
- ☐ Other, Please specify

If you selected Other, please specify:

What did the information tell you about being physically active during pregnancy?

How useful did you find this information? ✱ *Required*

- Not at all useful
- Not very useful
- Unsure
- Quite useful
- Very useful

Please add any comments if you wish :

Information you would like to receive

Would you like/have liked to receive any information about being physically active during pregnancy?

- ○ Yes
- ○ No
- ○ Not sure

Please tell us any other thoughts about this:

Where would you like/have liked to receive information from? Tick as many as you like.

- ☐ Online Forum
- ☐ Website
- ☐ Poster
- ☐ Diagram/Infographic
- ☐ Fitness Professional
- ☐ Family member
- ☐ Friends
- ☐ Leisure centre
- ☐ Magazine
- ☐ Leaflet
- ☐ Midwife & Health Professional
- ☐ Antenatal / Parentcraft classes

- [ ] GP Surgery
- [ ] Other, Please specify

If you selected Other, please specify:

How would you prefer information to be presented? Please tick as many as you like:

- [ ] Verbally one to one
- [ ] Verbally in a group or class
- [ ] Printed leaflet
- [ ] Online
- [ ] Posters
- [ ] Advertisement
- [ ] Diagram/infographic
- [ ] Other, please specify
- [ ] Other

Please let us know any other ways you would like information to be presented:

Guidelines

Are/were you aware of any formal recommendations/guidelines for physcial activity during pregnacy?

- Yes
- No
- Other

If yes, please feel free to give any opinions about this: *Optional*

Experiences of physical activity

Thinking about your current pregnancy or pregnancy for your youngest child - whichever is most appropriate:

How physically active did you consider yourself to be before becoming pregnant?

- Not at all active
- A little bit active
- Unsure
- Quite active
- Very active

Which of these best describe your physical activity during pregnancy?

- Not physically active during pregnancy
- Less physically active than before pregnancy
- As physically active as before pregnancy
- More physically active than before pregnancy

Which of the following statements would you most agree with about being physcially active during pregnancy?

- Very difficult to be active
- Quite difficult to be active
- Unsure
- Quite easy to be active

- Very easy to be active

When during your pregnancy were/are you most able to be physically active?

- 1st Trimester (0-12 weeks)
- 2nd Trimester (13-28 weeks)
- 3rd Trimester (29-40 weeks)

At what point during your pregnancy were/are you least able to be physically active?

- 1st Trimester (0-12 weeks)
- 2nd Trimester (13-28 weeks)
- 3rd Trimester (29-40 weeks)

What helped/helps you to be physcially active during pregnancy?

What has been the most helpful thing and why was it so useful?

What got/gets in the way of you being physcially active during pregnancy?

What has been the biggest hurdle and why did it make it so difficult or unappealing to be physically active?

How you feel about physical activity

How important is it to you to be physically active during pregnancy?

- Not at all important
- Not very important
- Unsure
- Important
- Very Important

Please let us know any reasons why:

How confident do/did you feel about being active during your most recent pregnancy?

- Not at all confident
- Not very confident
- Unsure
- Confident
- Very Confident

Please let us know any reasons why:

Top Tips

What three top tips would you give to a pregnant women who wants to be physically active during pregnancy ?

Is there anything else you'd like to share with us about your experiences?

Finish

Thank you for taking the time to participate in this online survey!

Please click the **Finish** button below to complete the survey.

If you would like any information about being physically active during pregnancy please follow this link to NHS Choices.

www.nhs.uk/conditions/pregnancy-and-baby/pages/pregnancy-exercise.aspx

For further information about this ongoing research please follow the link to the Moving Through Motherhood website.

www.movingthroughmotherhood.co.uk

Key for selection options

3.b - If you live in England, in what region do you live?

South West
South East
London
East England
West Midlands
East Midlands
Yorkshire and the Humber
North West
North East

7 - How many children do you have?

0
1
2
3
4
5
6
7
8
9
10+
