# Supplementary material for: Moving through Motherhood: Involving the Public in Research to Inform Physical Activity Promotion throughout Pregnancy and Beyond
Source: Int J Environ Res Public Health. 2021 Apr 23;18(9):4482. doi: 10.3390/ijerph18094482 (PMC8122923; doi:10.3390/ijerph18094482)
Supplement: Supplementary file 1 [file ijerph-18-04482-s001.zip › Supplementary file S2 Summary of qualitative data.pdf]

Supplementary file 1: Summary of qualitative data.

Many women responding to the survey felt PA was important, and keeping active was believed to be beneficial for general health and wellbeing for mum and baby, and to help with labour and recovery.

> *"For mine and my baby's wellbeing"*
>
> *"The more active I was the healthier I felt."*
>
> *"Maintaining health, to give strength and energy for birth and increased chance of a quick recovery after birth"*
>
> *"I wanted to stay fit for labour"*
>
> *"I was active all the way through both pregnancies and had easy births and recovered quickly both times"*
>
> *"Knowing that being fit will help during labour and in the early days of being a new mum"*

Benefits for mental health and mood, and limiting weight gain were also reported:

> *"I want to maintain my fitness levels generally, and don't want my fitness levels to drop during pregnancy as it would be more difficult to build back up after the birth and I feel so much better in myself when I maintain a good fitness level. Being physically active is good for my mental health and helps to reduce my anxiety."*
>
> *"I find it great for lifting my mood and energy levels - both if which were an issue during my pregnancy"*
>
> *"I find exercise really helpful for my mental wellbeing. It also makes me feel more in control and 'at one' with my body."*
>
> *"Also keeping fit helps me mentally and with my confidence …"*
>
> *"I have struggled with weight and health since I was young. I have tried to stay fit and healthy since I was 18 and I believe it will help me to recover after my pregnancy if I stay fit while pregnant"*
>
> *"Help stop too much weight gain. Good for my mental health. Physical fit for labour and recovery"*
>
> *"to help maintain a healthy pregnancy and weight, aiding a better birth by keeping fit."*

However, confidence with PA was challenged by lack of knowledge and information, uncertainty, pregnancy-related symptoms and medical issues, fear of judgement by others and concerns regarding safety.

Existing information can be vague, conflicting and confusing

Overall, advice reported by respondents who had receiving or reading information about being physically active in pregnancy appears to confirm that current information is vague and sometimes conflicting, with little reporting of information about what is safe, how much to do or how often. There is a wide range of information reported by respondents, highlighting that current advice relating to physical activity during/after pregnancy is not consistent or well understood by pregnant women or mothers.

For example, many respondents report receiving general advice to keep active or stay active:

> *"To keep active with gentle exercise"*

> *"It's good to stay physically active"*

This makes the assumption that the woman is already active before pregnancy. Advice not to start anything new and to take it easy is also unlikely to encourage anyone who is less active/inactive to try any physical activity when pregnant:

> *"Stay active during pregnancy but don't take on new forms of exercise - just keep going with what you were doing before."*

> *"I've faced a lot of misunderstandings about pregnancy and physical activity, being informed is very important but also being active because you like it. Somebody who is sedentary before won't chance a lot while pregnant, I think."*

Several women report conflicting advice to be active but not to overdo it, not to do anything strenuous or stick to light, gentle exercise, and other advice to do as much as you can:

> *"Some information was conflicting - keep exercise low impact (more stretching/ yoga/pilates type exercise) or do as much as you feel your body can take including weight-based training (listen to your body)."*

There is some evidence of respondents receiving advice that physical activity is good for mother and baby:

> *"It's important for both mother and baby's health. It's a way to feel more energetic throughout pregnancy. It will help resume a healthier lifestyle after birth/make for an easier birth."*

However, advice to avoid harm to the baby is also reported:

> *"I remember reading importance of avoiding certain exercise to avoid harm to baby."*

The perceived usefulness of information received by respondents was mixed. Negative experiences included lack of information or advice on what type of exercise to do and how strenuous this could be. Specific advice and detailed information was frequently lacking:

> *"I had some verbal guidance about activity levels but nothing specific or no signposting to groups etc"*

Conflicting advice meant it was difficult to know what to do, especially when getting any pregnancy-related physical symptoms:

> *"Information from different sources can be conflicting and therefore confusing. When the aches and pains of pregnant start it can be difficult to know whether to keep moving or stop and rest."*

> *"Getting different information is confusing and not very helpful"*

> *"Very wishy-washy advice from professionals I'd expect [advice] from during my pregnancy. I learnt more via my own research."*

Few women were aware of formal recommendations for PA in pregnancy, though this could be helpful to reassure them that PA is beneficial:

> *"I think the formal recommendations might have been the advice I saw on the poster (20 min walk per day?). I thought that a formal recommendation was a good idea, no matter what the amount of recommended activity was, as some people seem to think that you shouldn't do any physical activity during pregnancy (anecdotally, particularly 'older' generations, including my grandmother and mother in law!). A formal recommendation might indicate to pregnant woman that it is alright and even a good thing to be active during pregnancy."*

Few respondents reported awareness of advice for time, type, and intensity of PA per week in pregnancy:

> *"Healthy woman should get 150 hours a week of moderate intensity aerobic exercise"*

Many of those who indicated some awareness of formal recommendations/guidelines for PA often reported vague, non-specific advice:

> *"Gentle regular exercise unless told otherwise by your doctor or midwife"*

> *"All I was told was to do as much as felt comfortable to do!"*

> *"Don't start new fitness regimes when pregnant. Continue exercising at a similar level to pre-pregnancy"*

> *"It's quite vague "listen [to] your body" could be quite ambiguous. I would rather read something like "this type of [PA] should/shouldn't be done", "this or that type of exercise should be avoid[ed]""*

> *"Regular gentle exercise each week."*

Being active during/after pregnancy is not always easy

Several factors affected respondents' ability to be active during and after pregnancy. Common physical symptoms, such as tiredness, sickness and pain, as well as other medical issues and changing body shape were reported as hindering engagement in PA:

“Having the motivation to move/exercise when feeling sick and constantly drained of energy is hard.”

“Exhaustion! Often really struggle with energy levels for day to day without extra effort.”

“Tiredness and sickness in first trimester then the sheer size of me in my third trimester!”

“Sickness, fatigue and back pain. Lack of energy, the fact that I felt like a hippo waddling about!”

“Pelvic joint pain which became so severe towards the end that I could barely walk.”

“One of my biggest issues has be returning to sport post-pregnancies and the related issues with urine incontinence. Its an issue that isn't discussed much but women should be aware that realise they are not alone”

“Body shape changing made it hard to keep up with what to wear. Especially as shops do not stock maternity clothing and it is hard to judge sizing for online shopping.”

Some respondents reported that PA can actually help manage some common symptoms. For example, it may help improve energy levels:

“Couldn’t peel myself off the sofa but when I did I felt so much better.”

“Makes you feel good   Improves energy level”

“Persevere through fatigue - exercise helps with it”

“Even if you are tired try to do some physical activity regularly, as it actually energises you and helps with the tiredness.”

“Gentle walking is really relaxing for you. Helped beat tiredness and easily fits around your day.”

May reduce nausea:

“Feeling sick and bloated in the first trimester make it hard to go to the gym. although at times when I did go to a gym class I felt less nausea afterwards.”

“Sometimes exercise can actually Make symptoms Such as nausea better”

And can help ease pain:

“I keep my back problems in check with regular exercise.”

“A tiny bit of activity can make a big difference physically and mentally Gentle Exercise can help with pregnancy aches and pains”

However, it is important that PA information and advice acknowledges that being active is not easy for some women. Pressure to be active can have a negative impact if someone is struggling to participate in PA:

> *"I have severe [pelvic girdle pain]. I was a Personal Trainer. Now I can hardly walk. I can't continue exercising during pregnancy and now feel like a failure."*

> *"I was too unwell with severe pregnancy sickness to undertake physical activity. I tried to go to yoga but was sick. The only activity I've been able to undertake is walking, but now that is difficult due to pelvic pain. I felt a lot of pressure to be exercising when I really wasn't well enough to."*

> *"There is such a focus on praising women who are able to continue being active during pregnancy and to fit back into their pre pregnancy clothes within weeks of giving birth. If your pregnancy isn't like that it's easy to feel bad about yourself, beat yourself up and become depressed."*

> *"Please remember that many women would like to be active but can't. Pushing messages of potential harms from not exercising just makes them feel like more of a failure. There needs to be more awareness of how and why women need to be careful in pregnancy to avoid permanent damage."*

This highlights the importance of access to professional advice and support to adapt and modify PA according to individual needs for those women with specific concerns, for example, joint pain:

> *"Stop if you get any pain while exercising. Stop exercising and see a physio if you have any pain in your pelvic joints."*

> *"A physiotherapist may be able to help you with joint pain so you can continue to be as active as possible."*

<u>Feeling judged by others undermines confidence</u>

Many respondents felt judged and influenced by the views and opinions of others who did not think they should be active while pregnant:

> *"I got lots of comments from others about me cycling whilst pregnant- many thought i shouldn't be"*

> *"Peoples lack of knowledge of what pregnant ladies are capable of. The assumption that I cannot do anything was frustrating at times."*

> *"I only gave up cycling as my family and work were worried, I would have kept going otherwise."*

> *" [not] confident in 1st trimester due to societies views of exercising during pregnancy…."*

> *"I know that being active is very good for my health and in general for my pregnancy, however sometimes people around make me hesitate because of their*

comments "are you still doing this or that" , "take it easy", " you should take care", "are you sure it's safe""

"I found it annoying how many people felt the need to comment about me continuing to cycle pregnant - advising me to be careful and asking whether it was a good idea. The perceptions out there appear to be that you shouldn't exercise much."

Respondents advised that women should try to ignore comments or opinions of others, and felt that formal guidelines could help reassure pregnant women that PA is beneficial:

"Think of you and your baby, not what anyone else thinks of you."

"Don't compare yourself too closely to others"

"I thought that a formal recommendation was a good idea, no matter what the amount of recommended activity was, as some people seem to think that you shouldn't do any physical activity during pregnancy (anecdotally, particularly 'older' generations, including my grandmother and mother in law!). A formal recommendation might indicate to pregnant woman that it is alright and even a good thing to be active during pregnancy."

Having good support from partners/family/friends/other pregnant women for PA was useful for encouraging participation:

"I had very low energy levels for most of my pregnancy but a very high energy job so my partner encouraging me to go for walks with him during my lunch hour or at the weekend was very helpful."

"found a supportive community to allow me to continue"

"Go to somewhere where is pregnancy friendly or with someone else and you'll be fine"

"Doing exercise with other pregnant women"

"See if there is a friend or relative that you can go to a class with as this helps me to keep to a routine."

Information needs to address safety concerns

Concerns regarding safety of PA for mum and baby influenced decisions to undertake PA during pregnancy:

"I decided not to run during the first trimester as I'd had previous miscarriages and although I thought (and had been told by Midwife's) the miscarriage was not related to exercising I didn't feel confident about it."

"I was too scared to do normal exercises as I didn't know what was safe"

"Anxiety about health of the baby particularly in first pregnancy in the first trimester. I became inactive quickly and also put on too much weight. In the 3rd

trimester, I often ate a mars bar and a cold fizzy drink to get the baby moving as I was often worried about reduced fetal movement."

"Fear of injury to baby. Fear that certain activities (higher intensity activities) could cause damage/injury or worse."

"Worrying about the impact of exercise on my baby e.g. risk of miscarriage. Fear of hurting baby or miscarriage"

"1st trimester- fear of a miscarriage, even though I'm not sure here is evidence that there is any link."

"Guilt that it is harmful to baby"

"Scared of hurting the baby."

"Anxieties about hurting baby"

Respondents who were reassured that PA was safe felt more confident undertaking PA during pregnancy:

"I trusted the advice of my midwife and of peer reviewed research above all other sources of information and advice, and those advised overall that it was safe to continue to be active during pregnancy, so I felt confident to continue without causing any harm to the baby."

"I joined a pregnancy yogalates class which I loved, & did every week from around week 18 (when my sickness had stopped) right up until the night before I went into labour at 37+2. It was good to know the exercises were beneficial and safe."

As a result women would like information to include more detail about what is safe:

"[I] wanted to know what I could do whilst pregnant that wouldn't harm foetus."

"All I ever got told was 'keep active', but I also vaguely heard that there are maybe some activities that you shouldn't do, though this was never specified."

"I would have liked guidelines on the safe amount/type of exercise to do while pregnant."

"I would have liked to know what was beneficial/safe"

Engagement in PA was helped by support from trusted professionals, access to information about local opportunities and finding ways to fit PA into daily life.

<u>Professional advice is a source of reassurance and encouragement for PA</u>

PA advice received from health or fitness professionals was encouraging:

"I felt encouraged by the midwife to keep exercising, and messages about continuing to exercise being good"

"The advice from the midwife was the most useful as it was straight forward, meant that I could continue doing most of my usual physical activity if I felt like it, and I believed it was more likely to be reliable information than anything I read online (unless from reputable website, NHS website for example)."

"Pregnancy Pilates designed and led by very knowledgeable physio made me feel safe in how to exercise"

"Having a personal trainer which allowed me to know what I could and couldn't do."

Professional advice was particularly valued by those who had experienced pain:

"Guidance from physio. Specific pregnancy Pilates and physio appointment to help with pelvic pain."

"The care I received from the NHS antenatal physiotherapists was fantastic for my [pelvic girdle pain]. It meant I could continue my job until almost term and remain as active as was physically possible in both pregnancies."

<u>Information about local opportunities for pregnancy-related PA can support participation</u>

Some women would have liked further information about local classes or resources for pregnancy-friendly PA:

"It would be nice if information could be more specific or if there were places available to go for pregnant friendly workout."

"Midwife asked if I was exercising and suggested gentle exercise but I had no information about any pregnancy friendly related classes local to myself."

"It was very hard to find local options for pregnancy exercise classes. It all seemed to be yoga."

However, some respondents found that pregnancy classes were often inaccessible, for example due to location or cost:

"I looked into classes aimed at pregnant ladies in my area but found very little - had to travel 12 miles and pay parking to find wider selection. Thus not financially viable."

"Rural home location - limited availability of fitness classes in local area."

"I couldn't afford the classes as they were extortionate!"

"Perhaps having more classes available at more affordable prices would have helped"

Childcare arrangements may present additional challenges for some women for attending formal groups or classes:

“Without any regular childcare nearby I don't have the opportunities to exercise I would like”

“Able to continue with all my usual classes in first pregnancy as time was still my own. Much harder to find time when already have a child”

“ I worked full time whilst I was pregnant and had a two year old, so in the evenings I wouldn't be able to get out for a run (as I did in my first pregnancy), as I had to look after my elder child when my husband was working late (which was a lot!).”

For women who are unable to attend classes, information about alternative options for exercising at home may be helpful:

“Option to squeeze in at home exercises […] using exercise videos, yoga videos.”

“YouTube yoga videos during third trimester.”

“dvds designed specifically for pregnancy. [dvds have been most helpful] as I knew they were safe.”

<u>It is helpful to fit activity into daily life</u>

Beyond formal exercise instruction, many women found ways to fit activity into their daily life, particularly when taking part in formal activities was challenging:

“Cycling to work - easy and time-efficient but I’m also convinced it contributed to me having an ‘easy’ birth”

“Integrating activity into daily life as much as possible e.g. walking to appointments”

“I am in the habit of walking everywhere, so I kept this up for as long as possible. It also helped clear my head if I was feeling anxious.”

“Undertaking everyday activities like walking from a to b, and recognising this as exercise - I have been too unwell to do formal classes or organised activities.”

“Walking places that I needed to get to was a good way of staying active, when I couldn't do some of the more strenuous activity I was doing before pregnancy.”

Recognising that PA doesn’t have to mean formal fitness can be helpful:

“Try to remain as active in day to day activities as possible without feeling you have to participate in any specific 'fitness activity'”

“Just get outside and walk. Look around you and enjoy the sounds and sights as you go.”

“You don't need to go to a gym, a brisk walk in the fresh air is just as good.”

Some women found that keeping up a routine enabled participation in regular PA:

"The routine I already had I tried to keep up - swimming and yoga once a week"

"A routine (took a yoga class at the same time every week)"

"Obviously everything became more hard work as pregnancy went on but being used to doing a little everyday and cycling to work helped."

While childcare presented a challenge for structured PA for some respondents, others found having other young children enabled them to be active more generally:

"Having a young child to get out and about with"

"Having a younger child to keep me busy and who needs constant entertainment"

<u>Encourage little and often and be gentle with yourself</u>

Respondents provided several tips for other women for staying active. These included taking small steps and setting small goals to try and keep moving little and often, particularly as pregnancy progresses:

"Small steps - take the stairs, take the long route to the shop. Easy things to keep you moving."

"Think about what you already do and try to keep doing it Accept that you might have to modify what you do to accommodate your changing body but try not to stop altogether Set small goals"

"Do what feels right for you. It's better to do some physical activity than nothing!"

It was considered important to keep within personal limits, and to acknowledge the physical and emotional changes that might impact on PA during/after pregnancy:

"Be gentle with yourself Get some exercise clothes that will be comfortable and make you feel confident"

"It is important to live an active lifestyle generally but there shouldn't be pressure to exercise when feeling unwell as long as you are living a moderately healthy lifestyle with diet, sleep and adequate movement throughout the day"

"Be realistic Be kind to yourself"

"Find something you enjoy. Get a friend (or dog!) to go with you. Don't feel too bad if you just can't face it one day, just start again the next day."

"Do it. Make the time for it. If your too tired don't beat yourself up about it."

"Fit it into your routine Don't sweat the exhausted days"

"1. Join a class or group where you can get instruction. 2. Don't be afraid to carry on if you were already active. 3. Don't feel bad if you're too tired to exercise; you're growing a human!"

PA was important to some respondents as it allowed them to focus on themselves, when a lot of pregnancy is focused on the baby:

"… it's time for myself"

"Felt part of me, when everything else was baby"

"Find something you enjoy and use it as me time"

<u>Positive experiences that helped to be active in pregnancy</u>

Table: Factors identified within open text responses as being helpful for PA in pregnancy

What helps to be physically active in pregnancy?	Example supporting evidence
Minimal pregnancy-related physical symptoms, e.g. tiredness, sickness, pain	"The fact that my symptoms have not been too bad and that I have felt up to being physically active."<br>"Not feeling tired/nauseous"<br>"Struggled in first trimester with nausea, sickness and fatigue, and third with fatigue, hip and back pain. In the middle I felt ok and that was when I could be active comfortably"<br>"I have not suffered too much with symptoms and that has helped a lot. If I had sickness or a lot of pain I would have found it much more difficult to be active."<br>"Lack of fatigue"
Having other children	"Having a young child to get out and about with"<br>"The thing that made me physically active was the need to walk to get to work etc or to look after the toddler."<br>"Getting a dog & a toddler ... they need excercise & play time whether I feel like it or not!"<br>"Having a younger child to keep me busy and who needs constant entertainment"
Having access to childcare	"Childcare for my children so I could go to classes."<br>"Money and supportive partner as I could afford to go to exercise classes while my partner took care of our other children."
Specialist advice and support	"Professional guidance- reassuring u and baby r benefiting"<br>"Having a personal trainer which allowed me to know what I could and couldn't do."<br>"The trainers in my gym helping me out on what I should and shouldn't be doing"<br>"Supportive fitness person leading exercise group"
Support from social network	"Doing exercise with other pregnant women"<br>"Supportive and active partner"<br>"Supportive family and friends"<br>"Support from family and friends and maintaining those throughout the pregnancy"

	"Online community - introducing specified exercises for pregnant woman."
Knowing what is safe	"Yoga and aqua natal were useful because they were both gentle exercise and helped with the pain in back and hips. It was also reassuring to know because aimed at pregnancy they were "safe""<br>"Knowing that something was safe. Peace of mind."<br>"Knowing what was safe to do."
Dog walking	"Have a dog so no choice but to walk him every day"<br>"Having dogs to walk every day meant I was out walking with them a lot"<br>"I was also walking 2 hours a day, right up until the day I went in to labour...because I had a dog that needed walking"<br>"having a dog to walk, you had to walk no matter how you felt"
Home exercise options	"Option to squeeze in at home exercises when not feeling tired using exercise videos, yoga videos."<br>"…dvds designed specifically for pregnancy."<br>"Pilates DVD recommended by physiotherapist treating me for SPD"
Active transport/travel	"Cycling to work - easy and time-efficient but I'm also convinced it contributed to me having an 'easy' birth and very little pelvic floor issues afterwards."<br>"The thing that made me physically active was the need to walk to get to work etc"<br>"Integrating activity into daily life as much as possible e.g. walking to appointments"<br>"Not having a car and needing to walk everywhere."<br>"Undertaking everyday activities like walking from a to b, and recognising this as excercise - I have been too unwell to do formal classes or organised activities."<br>"Walking places that I needed to get to was a good way of staying active, when I couldn't do some of the more strenuous activity I was doing before pregnancy."<br>"I cycled to work every day until I left which was six weeks before baby due."
Routine	"The routine I already had I tried to keep up - swimming and yoga once a week"<br>"A routine (took a yoga class at the same time every week)"
Money	"Money […] as I could afford to go to exercise classes..."<br>"Free swimming in Bristol pools"<br>"swimming was free and relaxing and took the weight off. "
Local opportunities	"Swimming pool nearby"<br>"Availability of local pregnancy classes, such as a pregnancy yoga class which I did."<br>"Supportive family and friends and opportunities locally."<br>"Suitable activities available at local leisure centre, at times I can make."
Time	"Joining a Turtle Tums class where the exercise was adapted and also at a suitable time to fit round toddler"<br>"Time to be active (maternity leave)"

	"Able to take time to look after yourself"
Pre-pregnancy activity	"Already exercising regularly before I became pregnant"<br>"Being used to activities that I was able to continue or adapt to pregnancy (yoga and cycling)."<br>"Carrying on gym classes you did before your pregnancy that are not too taxing"<br>"being sporty before pregnancy."<br>"The fact that I was already physically active beforehand."
