# Supplementary material for: Moving through Motherhood: Involving the Public in Research to Inform Physical Activity Promotion throughout Pregnancy and Beyond
Source: Int J Environ Res Public Health. 2021 Apr 23;18(9):4482. doi: 10.3390/ijerph18094482 (PMC8122923; doi:10.3390/ijerph18094482)
Supplement: Supplementary file 1 [file ijerph-18-04482-s001.zip › Supplementary file S3 example like me stories.pdf]

Moving through Motherhood

‘Like me’ stories

Example 1 – safety concerns after miscarriage

**Template item**	**Selected information**
Background	32 year old mother, previous anxiety issues, previous miscarriages
Concerns	I was too scared to do normal exercises as I didn't know what was safe, and I was worried about the impact of exercise on my baby because of previous miscarriages.
Positive experience/helpful advice	My midwife reassured me it is safe to be active.<br>Walking places that I needed to get to was a good way of staying active, especially when I couldn't do some of the more strenuous activity I was doing before pregnancy.
Tips for other women	It is important to keep active during pregnancy for yours and the babies well being.

Example 1 illustration – ‘Priya’

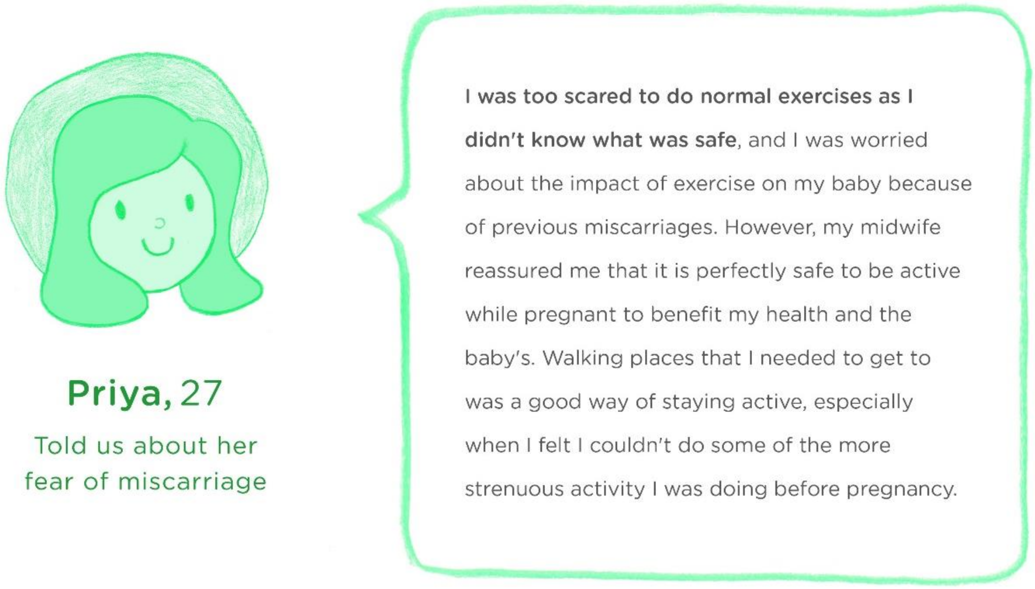

Example 2 – nausea and tiredness

**Template item**	**Selected information**
Background	Nausea, tiredness
Concerns	Having the motivation to move/exercise when feeling sick and constantly drained of energy is hard
Positive experience/helpful advice	Even though I felt sick I tried to think about what physical activity I already did and tried to keep doing it. I found it lifted my mood and energy levels, and I even noticed that exercise can actually sometimes make as nausea better!

Tips for other women	Accept that you might have to modify what you do to accommodate your changing body but try not to stop altogether. Set yourself small goals.

Example 2 illustration – 'Sally'

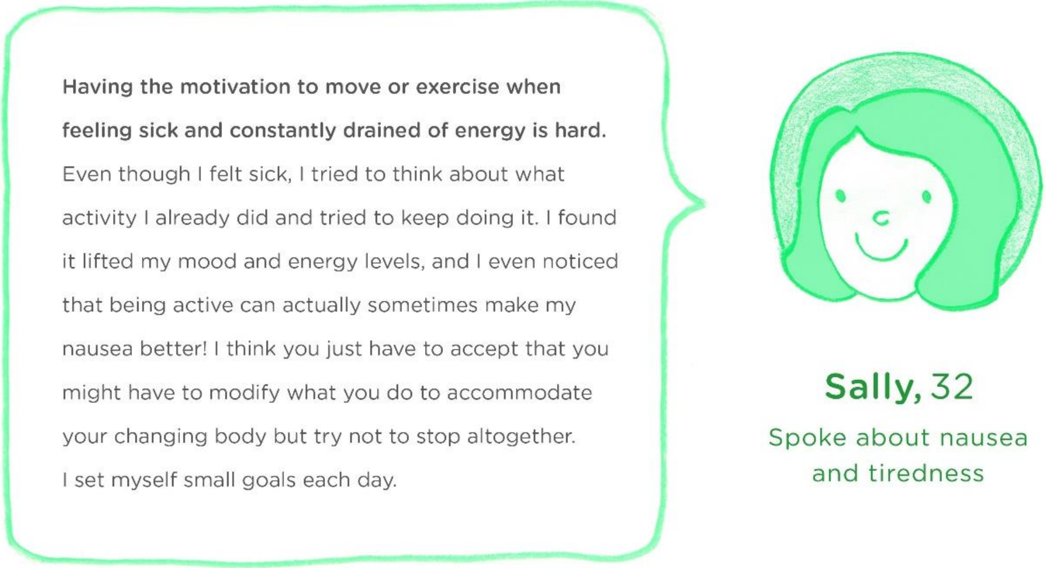

Example 3 – pelvic pain

Template item	Selected information
Background	pelvic pain
Concerns	I experienced pelvic girdle pain which became severe towards the end. I worried that exercise would make it worse, and all the advice about exercise made me feel like a bit of a failure because I wanted to be active but I couldn't.
Positive experience/helpful advice	My midwife referred me to a physiotherapist, who gave lots of good advice and support.
Tips for other women	Be realistic and kind to yourself. If exercise is painful, stop and ask to see a physiotherapist.

Example 4 – timing/duration of physical activity

Template item	Selected information
Background	150 minutes seems like a lot<br>Has another child already<br>Experiences anxiety
Concerns	I saw a poster that said I should be doing 150 minutes of exercise a week. This seemed like loads and made me feel quite anxious that I wasn't doing enough, especially as I had a toddler to look after as well.

Positive experience/helpful advice	My friend told me that everyday activities like walking count as exercise, and this was really helpful. I realised that having a young child to get out and about with kept me moving for at least half an hour every day!<br>I am in the habit of walking everywhere, so I kept this up for as long as possible. It also helped clear my head if I was feeling anxious.
Tips for other women	Even if you can only face going for a walk - do it - you will feel better afterwards Try to give it a go and if you can only last for 15 minutes it's 15 minutes more than you would have done otherwise. Take the stairs, or the long route to the shop. Easy things to keep you moving.

Example 5 - intensity

**Template item**	**Selected information**
Background	Does not understand what is meant by moderate intensity activity, therefore not sure what to do
Concerns	Most websites/pregnancy apps advised doing moderate intensity activity but I had no idea what that meant!
Positive experience/helpful advice	Someone at the leisure centre explained that this means that you are breathing a harder and feeling warmer when you are doing the activity, but you can still have a conversation with someone. This could be going for a brisk walk or doing other exercise e.g. walking, jogging, cycling, swimming. This reassured me that cycling to work was moderate intensity. I found this easy and time-efficient but I’m also convinced it contributed to me having an ‘easy’ birth!
Tips for other women	Trying to remain as active as possible in your usual day to day activities can mean you are doing ‘moderate intensity activity’ without feeling you have to take part in any specific 'fitness activity'.
